# Insertion of the Liquid Crystal 5CB into Monovacancy Graphene

**DOI:** 10.3390/molecules27051664

**Published:** 2022-03-03

**Authors:** Paul A. Brown, Jakub Kołacz, Sean A. Fischer, Christopher M. Spillmann, Daniel Gunlycke

**Affiliations:** 1 Chemistry Division, United States Naval Research Laboratory, Washington, DC 20375, USA; paul.brown@nrl.navy.mil (P.A.B.); safischer012@gmail.com (S.A.F.); 2 Center for Bio/Molecular Science and Engineering, United States Naval Research Laboratory, Washington, DC 20375, USA; jakub.kolacz@nrl.navy.mil (J.K.); christopher.spillmann@nrl.navy.mil (C.M.S.)

**Keywords:** liquid crystal, graphene, defect, monovacancy

## Abstract

Interfacial interactions between liquid crystal (LC) and two-dimensional (2D) materials provide a platform to facilitate novel optical and electronic material properties. These interactions are uniquely sensitive to the local energy landscape of the atomically thick 2D surface, which can be strongly influenced by defects that are introduced, either by design or as a byproduct of fabrication processes. Herein, we present density functional theory (DFT) calculations of the LC mesogen 4-cyan-4′-pentylbiphenyl (5CB) on graphene in the presence of a monovacancy (MV-G). We find that the monovacancy strengthens the binding of 5CB in the planar alignment and that the structure is lower in energy than the corresponding homeotropic structure. However, if the molecule is able to approach the monovacancy homeotropically, 5CB undergoes a chemical reaction, releasing 4.5 eV in the process. This reaction follows a step-by-step process gradually adding bonds, inserting the 5CB cyano group into MV-G. We conclude that this irreversible insertion reaction is likely spontaneous, potentially providing a new avenue for controlling both LC behavior and graphene properties.

## 1. Introduction

Liquid crystal (LC) on atomically thin 2D interfaces results in unique behaviors such as surface-induced phase transitions [1], tilt angle control [2], selective alignment [3], and ion trapping [4]. While research on integrated LC-2D material systems is still in its infancy, their properties have been exploited to image and characterize 2D materials using LC to optically transduce surface defects [5], domains [6,7], degradation [8] and the number of 2D layers [2]. Conductive 2D materials such as graphene have also been demonstrated as a single-component electrode and alignment layer [9,10]. The large effect of atomic-level interactions demonstrates the importance of 2D surfaces on further miniaturization of LC electrooptic devices, leading to smaller form factors, faster response times [11] and new behaviors.

Defect engineering of 2D materials is increasingly becoming feasible on the atomic scale [12,13,14]. Atomic-scale control is a critical challenge for nanoscale development of new materials for nascent technologies. Research on 2D materials have expanded rapidly [15,16,17,18] and influenced electronics [19,20], catalysis [21,22,23], energy storage [24], batteries [25], topotronics [26,27], sensing [28] and photonics [29,30,31,32]. Moreover, 2D materials, including graphene, provide a large surface area for anchoring of atoms or molecules introduced into the system. Furthermore, advances in nanoscale patterning and defect engineering raise the prospect of incorporating and controlling liquid crystals on such surfaces [33,34]. 2D materials could thus facilitate exceptional binding of mesogen compounds, especially compounds whose chemical structure resembles the underlying 2D material itself as such compounds could, e.g., bind through π-π stacking [35,36,37,38]. In addition, we expect that defects will play a central role in future applications, as they can provide both control and anchoring sites for absorbates.

Defects often arise naturally in 2D materials during fabrication, including monovacancies, divacancies, Stone-Thrower-Wales defects, line defects, grain boundaries and impurities [39,40,41,42,43,44,45,46,47,48,49]. Herein, we focus on monovacancy graphene (MV-G), as graphene is a well-studied and easily fabricated 2D material and the monovacancy is a common and simple intrinsic point defect, acting as a reactive center for binding atomic or molecular matter [21,22,23,48,50,51,52]. Despite this, the attention in the field has so far focused on the properties of LC on pristine nanosheets [36,37,38], with little consideration of the potential reactivity of defects. 

In this article, we present DFT calculations exploring the possibility that 5CB chemically reacts with a monovacancy in MV-G and under what circumstances such reactions take place. To probe this reactivity, we consider MV-G with three 5CB anchoring alignments: planar, homeotropic and pre-tilt. Our analyses include nudge elastic band (NEB) calculations to determine the reaction pathway and projected density of states (PDOS) calculations to identify key atoms involved in the insertion process. Lastly, we discuss the source of the reactivity in homeotropically aligned 5CB. 

## 2. Results and Discussion

### 2.1. 5CB/MV-G Interaction

**Figure 1:**
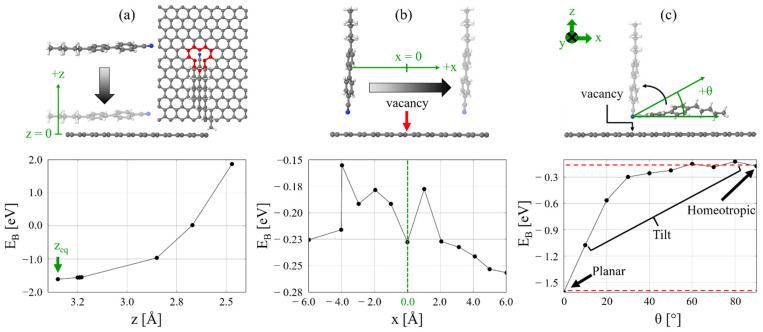
Binding energy scans. (**a**) Planar scan starting at the equilibrium distance z_eq_. (**b**) Homeotropic scan relative to the monovacancy position. (**c**) Pre-tilt scan with dashed red lines marking the planar and monotonicity of the binding energy.

We begin by considering the equilibrium structure of 5CB adsorbed onto MV-G in the planar alignment. In this case, the equilibrium distance is 3.34 Å with a binding energy of −1.60 eV (Figure 1a). The binding energy of 5CB onto MV-G is 0.29 eV lower than that for the corresponding 5CB binding energy −1.31 eV onto graphene without the monovacancy [38]. The adsorption energy can be viewed as a molecular analog to surface anchoring energy for the LC, suggesting a stronger surface anchoring energy in the presence of the monovacancy.

To probe the possibility of chemical binding, we explored 5CB in the planar orientation being compressed toward the monovacancy. The cyano substituent is oriented with the cyano-carbon above two of the carbon sites immediately adjacent to the Jahn–Teller-distorted monovacancy and the cyano-nitrogen above the third adjacent monovacancy carbon atom. The reason for this orientation is that the cyano group, which is a highly polarized substituent and responsible for the dipolar character of 5CB, exposes the partially positive cyano-carbon to the dangling bonds of the monovacancy carbon atoms, maximizing the likelihood to induce a reactive event. This commonly occurs in reductive elimination reactions of cyano functional groups [53,54]. As is shown in Figure 1a, however, the computed binding energy increases monotonically as 5CB approaches, suggesting that there is no chemical bonding between the 5CB and the monovacancy in the planar orientation. Not shown in this figure is that the compression of 5CB onto MV-G also induces stress, causing a single monovacancy carbon atom to protrude out-of-plane, away from the 5CB. The two Jahn–Teller carbons flex toward 5CB, further indicating that the possibility of planar attachment to the monovacancy is unlikely. 

Next, we consider the homeotropic alignment to probe for potential reactivity (Figure 1b). Overall, a similar pattern of the binding energetics emerges for homeotropic alignment of 5CB compared to the planar case though with some noticeable differences. The lowest binding energy is −0.26 eV when the lateral in-plane distance between the 5CB cyano substituent and the monovacancy is 6.0 Å. We also observe a relatively low adsorption energy between −4.0 Å and 2.0 Å. Above the monovacancy, the binding energy is −0.23 eV. Though the planar binding energy is lower than the corresponding the homeotropic binding energy, steric effects make chemical binding unlikely. Instead, we expect that the homeotropic alignment is more conducive to chemical binding.

We also performed an angular scan between the planar and homeotropic orientations. As Figure 1c shows, the binding energy increases linearly between 0–20° and then saturates at approximately −0.20 eV approaching the homeotropic state. This analysis indicates that for partial coverage of 5CB on the graphene surface, approximately 1.4 eV is required for the molecule to reach homeotropic alignment above the monovacancy. However, because the binding energy saturates near 30°, homeotropic alignment could also easily be reached for a 5CB molecule approaching MV-G within a large range of angles around normal incidence.

**Figure 2:**
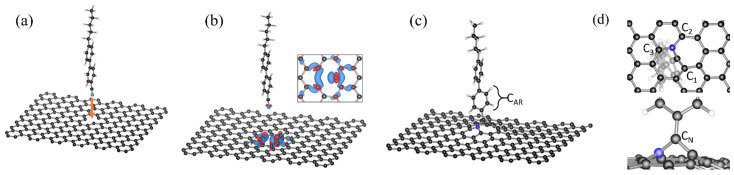
(**a**) 5CB homeotropically aligned above the monovacancy in MV-G. (**b**) Spin-polarized density of 5CB/MV-G with the inset showing a planar view of the corresponding density for the Jahn-Teller-distorted MV-G. (**c**,**d**) Images of the inserted 5CB molecule. The cyano-nitrogen (N) has filled the monovacancy and reestablished the hexagonal structure of graphene (upper panel in (**d**)), with the exception that the cyano-carbon (C_N_) has formed a bridge between N and the carbon site (C_1_). The bridge chemically binds the aromatic carbons (C_AR_) and the rest of the 5CB molecule to the surface.

To understand the potential reactivity between 5CB and the carbon vacancy site, 5CB is placed in the homeotropic state directly above MV-G and allowed to relax into the monovacancy site (Figure 2a). The initial electron density (Figure 2b) is independently localized on the Jahn–Teller distorted monovacancy and on the cyano head of the 5CB molecule. As the molecule approaches the surface, the cyano head inserts itself into the defect, stabilizing the structure and causing the biphenyl core to twist (Figure 2c). The final structure exhibits one hexagonal and two non-hexagonal rings (Figure 2d) [55,56] that tilt out of the plane at the cyano-nitrogen (N), cyano-carbon (C_N_) and first carbon (C_1_).

**Figure 3:**
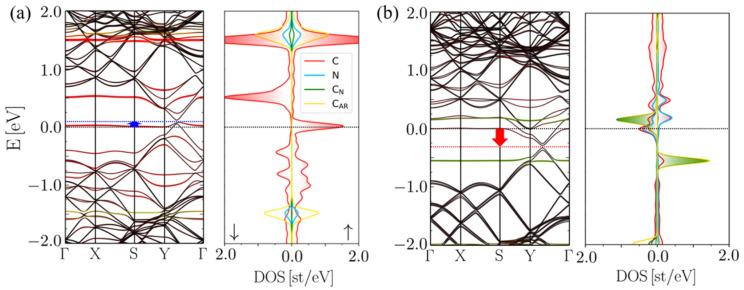
Spin-polarized band structures and density of states plots of (**a**) planar and (**b**) homeotropically inserted 5CB/MV-G. The blue and red arrows in the band structures indicate p- and n-doping of the graphene, respectively. Spin up (positive) and spin down (negative) are highlighted in the right panels.

An analysis of the spin-polarized band structures (Figure 3a) shows that the presence of 5CB in the planar state near a monovacancy has only a small effect on graphene’s electronic properties. The slight p-doping of the graphene, as is shown by the shift in the Dirac point, occurs due to Jahn–Teller distortions in MV-G and is observed even without the presence of 5CB [57,58]. The small HOMO-LUMO energy gap is also consistent with values for MV-G [59]. The DOS exhibits symmetry in the spin states of the 5CB molecule (N, C_N_ and C_AR_ peaks), which means the molecule remains in a non-magnetic state with a DOS similar to that of 5CB on pristine graphene [38], while the MV-G itself is in a magnetic state. 

In the case where homeotropic 5CB comes into contact with the monovacancy, the insertion reaction results in a mixing of the orbitals that induces a magnetic state in the 5CB constituent (Figure 3b). This is evidenced by the different DOS peaks localized at the 5CB sites for spin up and down. The magnetic moment is around 1.5 µ_B_. The −260 meV shift in the Dirac point results in n-doped graphene. Furthermore, the band structure of inserted 5CB/MV-G shows strong orbital mixing in bands near the Fermi energy from the cyano-carbon, cyano-nitrogen, aromatic carbons and monovacancy carbon atoms. 

### 2.2. Insertion Reaction

**Figure 4:**
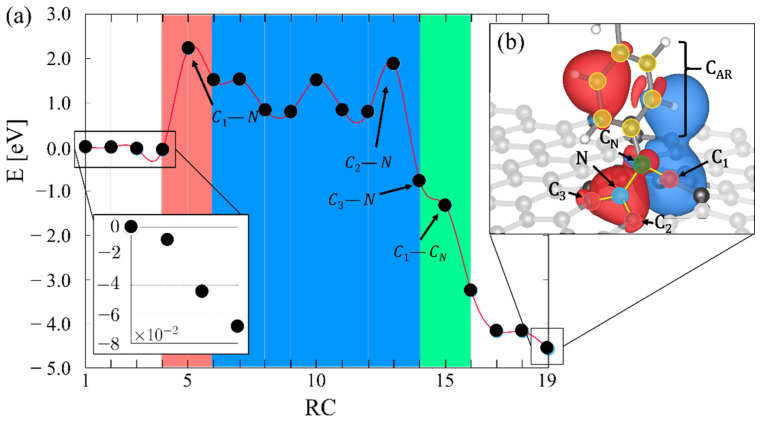
(**a**) Reaction path for the reaction coordinate (RC) of 5CB homeotropic insertion. The colored background marks the important chemical events along the reaction path beginning with insertion (red), tetrahedral formation (blue) and cyano-carbon reorganization (green). Inset shows the initial approach. (**b**) Spin-polarized charge density difference (Δρ [bohr^−1^]) of the final state.

An NEB calculation provides insight into the reaction path for 5CB insertion into MV-G. The energy analysis is shown in Figure 4a (The corresponding structures and PDOS can be found in the Supplementary Materials in Figures S1 and S2, respectively). The first four data points (reaction coordinates 1 through 4) show the total energy during the initial approach of 5CB to the monovacancy, starting at a distance of 5.25 Å and ending at 1.61 Å above the nanosheet. Note that all our calculations in this region produce non-magnetic solutions. Any potential magnetic solution, however, is expected to be close in energy, as past DFT calculations on graphene with a monovacancy without 5CB suggests that the largest energy difference between a magnetic and the non-magnetic solution is of the order 3 × 10^−2^ eV [60]. As 5CB reaches its physisorbed state at 1.61 Å above the monovacancy the overall energy lowers by nearly −7 × 10^−2^ eV (Figure 4 inset). The lowered energy suggests that initial 5CB insertion into the monovacancy is stabilized.

The initial barrier along the reaction path is approximately 2.2 eV (Figure 4a, red shading), which is consistent with reported energy barriers for insertion of mon- and di-atomic species into monovacancy graphene [61,62]. This region marks the initial insertion of the cyano head into the monovacancy. The energy of the system remains high as the N site bonds in turn to C_1_, C_2_ and C_3_, as shown by the blue region in Figure 4a. Once the N bonding is complete, the four sites N, C_1_, C_2_ and C_3_ form an sp^3^-hybridized tetrahedral structure that lowers the energy significantly. The green region of Figure 4a highlights the steps where C_1_ bonds first to C_N_ and then detaches from the nitrogen, creating a bridge between C_1_ and N. This stage allows all relevant sites, including N, C_N_ and C_1_ to become sp^2^-hybridized, which results in a further energy reduction. The last step of the reaction twists the biphenyl of the 5CB, reducing the delocalization of charge in the core of the molecule [37]. The final structure is 4.5 eV lower in energy than the initial structure and the residual spin-polarized charge density difference in Figure 4b includes sites across the phenyl ring (aromatic carbons in yellow) extending to the monovacancy in MV-G.

**Figure 5:**
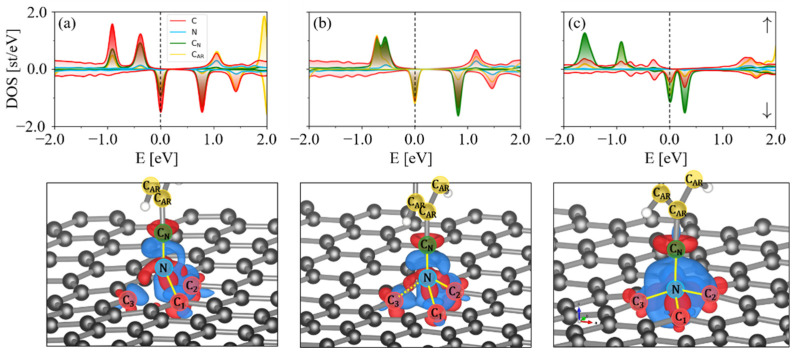
PDOS and structures during tetrahedral nitrogen formation from Figure 3. (**a**) C_1_-N bond formed at RC = 5. (**b**) C_2_-N bond formed, with a partial C_3_-N bond beginning to form at RC = 13. (**c**) C_3_-N bond fully formed at RC = 14. The color scheme is as follows: (red) vacancy carbon atoms, (blue) cyano-nitrogen (N), (green) cyano-carbon (C_N_) and (yellow) aromatic carbons (C_AR_). The spin density difference for (**a**–**c**) is shown beneath the PDOS (Δρ [bohr^−1^]), respectively.

To highlight the salient steps in the reaction path, Figure 5 shows the PDOS of the monovacancy carbons, cyano-substituents and aromatic carbons at certain steps in the reaction. First, charge mixing appears between the cyano substituent and the monovacancy (Figure 5a), initially forming an N-C_1_ bond. At this stage, the charge density is localized around the carbon atoms in the monovacancy. After the first bond is formed, there is a peak in the DOS at the fermi energy stemming from the aromatic carbons in the 5CB molecule (yellow peak in Figure 5b). The appearance of this peak suggests that delocalized charge in the phenyl ring plays a role in the charge reorganization that leads to N-C_3_ bond formation. Finally, when the last tetrahedral nitrogen bond is formed at N-C_3_, the dominant DOS contribution near the Fermi energy is a result of C_N_ (Figure 5c). The tetrahedral structure is evident. At this point, there is only a single bond holding the cyano-group together. Note the increased N-C_N_ bond length in Figure 5c compared to that in Figure 5b. This sets the stage for the final bond reorganization that connects the under-coordinated C_N_ with the monovacancy at C_1_, leading to the final structure shown in Figure 4b.

## 3. Materials and Methods

The structures herein were investigated using the projector augmented wave (PAW) method within the Vienna Ab Initio Simulation Package (VASP 5.4.4) [63,64,65,66]. All calculations are spin-unrestricted and performed using the Perdew–Burke–Ernzerhof (PBE) functional combined with the DFT-D3 van der Waals correction [67,68,69]. To provide sufficient space for 5CB on MV-G, an 8 × 6 orthorhombic supercell with a 34 Å out-of-plane vacuum space is used. These comparisons converge as cell sizes increase; thus, we expect small changes in physical properties between the presented orthorhombic cell and the more commonly used hexagonal cell.

Each system is optimized with a cutoff energy of 600 eV and a force tolerance of 0.01 eV/Å. Geometric optimizations are performed at Γ. These optimizations are followed by band structure, PDOS and binding energy calculations using a 15 × 15 × 1 Γ-centered *k*-grid. The binding energy ($E_{B}$) is herein defined as

(1) $$E_{B}=E_{5CB/MVG}-\left(E_{5CB}+E_{MVG}\right),$$

where $E_{5CB/MVG}$, $E_{5CB}$ and $E_{MVG}$ are ground-state energies of the supercell, 5CB and MV-G, respectively [37]. For the homeotropic scans, 5CB degrees of freedom are free to relax, while MV-G degrees of freedom are frozen. In the planar alignment, 5CB degrees of freedom are frozen to avoid deflection of the molecule away from MV-G and the boundary carbon atoms of MV-G are frozen to prevent nanosheet repulsion, i.e., the nanosheet being pushed down along the negative *z*-axis as 5CB is brought inside equilibrium distance. For the NEB calculations, we use 19 total images and include the spin-polarized charge density difference for important transitions along the reaction path. See Supplementary Material for the entire set of NEB images and PDOS. The spin-polarized charge density difference is determined from

(2) $$\Delta \rho =\rho_{5CB/MVG}-\left(\rho_{5CB}+\rho_{MVG}\right),$$

where $\rho_{5CB/MVG}$, $\rho_{5CB}$ and $\rho_{MVG}$ are the total spin-polarized charge densities for the composite system, 5CB and MV-G, respectively.

## 4. Conclusions

Using DFT, we investigated configurations that could lead to 5CB implantation into a monovacancy defect in MLG. Given the importance of orientation for initiating a reaction, we found homeotropic alignment of 5CB enabled spontaneous insertion into the monovacancy. The reaction takes several salient steps: (1) an initial bond with a monovacancy carbon pulls the 5CB toward the sheet; (2) the cyano-nitrogen forms a tetrahedral bond with the three carbanions in the monovacancy; (3) the resulting under-coordinated cyano-carbon is pulled further into the monovacancy; (4) one of the three monovacancy carbons unbinds the cyano-nitrogen and binds with the cyano-carbon. From the DOS, we can see that the process is mediated by the conjugated biphenyl core of the molecule. The resulting structure stabilizes the monovacancy by inserting both the cyano-carbon and cyano-nitrogen into the sheet, with the biphenyl core aligned normal to the surface. This allows charge to flow between the sheet and the 5CB, shifting the Fermi energy of the graphene.

It is important to note that the homeotropic alignment of the 5CB molecule on MV-G is dissimilar to the preferred planar orientation on pristine graphene [37]. In the LC phase, this would cause large geometric frustrations in 5CB, which could lead to a weakening of the anchoring energy, a degenerate pretilt at the surface or defects in the LC orientation. On a micro-scale, defect engineering of LC systems has already revealed novel self-assembly, memory effects and control of flow and colloidal motion [70]. Intelligent design of defects such as monovacancies in 2D surfaces would yield a new testbed for examining LC behavior.

Furthermore, our results indicate that defects can play a key role in the interfacial chemistry of LC-based devices incorporating 2D nanosheets. Future work at the intersection of surface vacancies and LC could have broadened significance with respect to patterned defects on 2D nanosheets for application-driven covalent surface functionalization, which could raise new questions concerning tailored interfacial interaction for specific applications.

## Data Availability

The data presented in this study are available on request from the corresponding author. The data are not publicly available due to U.S. Department of Defense technical information controls.

## Supplementary Materials

The following supporting information can be downloaded online, Figure S1: NEB structures for 5CB/MV-G insertion reaction, Figure S2: NEB PDOS for 5CB/MV-G insertion reaction.

## Sample Availability

Not available.
