# Supplementary material for: Insertion of the Liquid Crystal 5CB into Monovacancy Graphene
Source: Molecules. 2022 Mar 3;27(5):1664. doi: 10.3390/molecules27051664 (PMC8911687; doi:10.3390/molecules27051664)
Supplement: Supplementary file 1 [file molecules-27-01664-s001.zip › molecules-1576238-supplementary.pdf]

Supplementary Material for

Article

Insertion of the Liquid Crystal 5CB into Monovacancy Graphene

Paul A. Brown, Jakub Kołacz, Sean A. Fischer, Christopher M. Spillmann and Daniel Gunlycke *

U.S. Naval Research Laboratory, Washington DC 20375, USA
* Correspondence: daniel.gunlycke@nrl.navy.mil

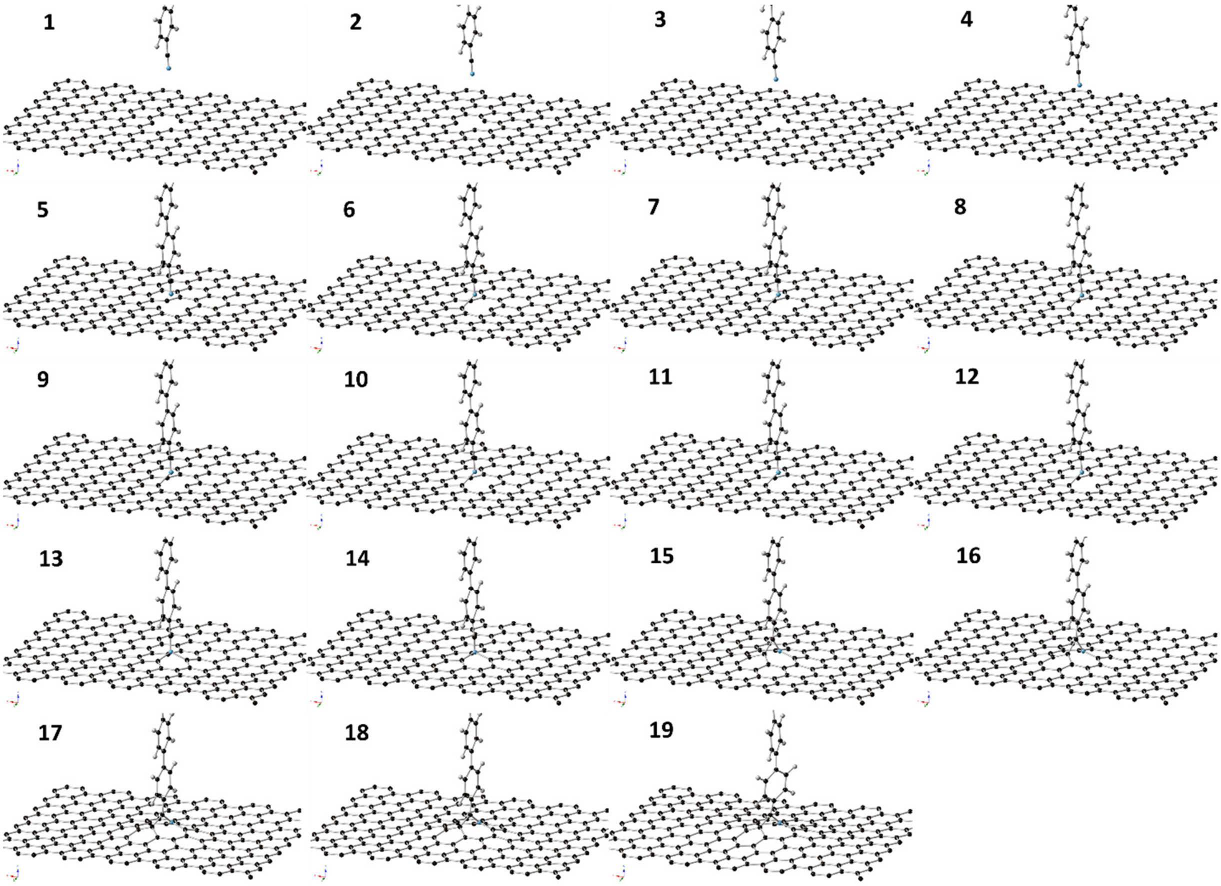

Figure S1. Atomic structures for the 19 reaction steps generated by the nudge elastic band method for the process of chemical insertion of 4-cyan-4'-pentylbiphenyl (5CB) into a graphene monovacancy.

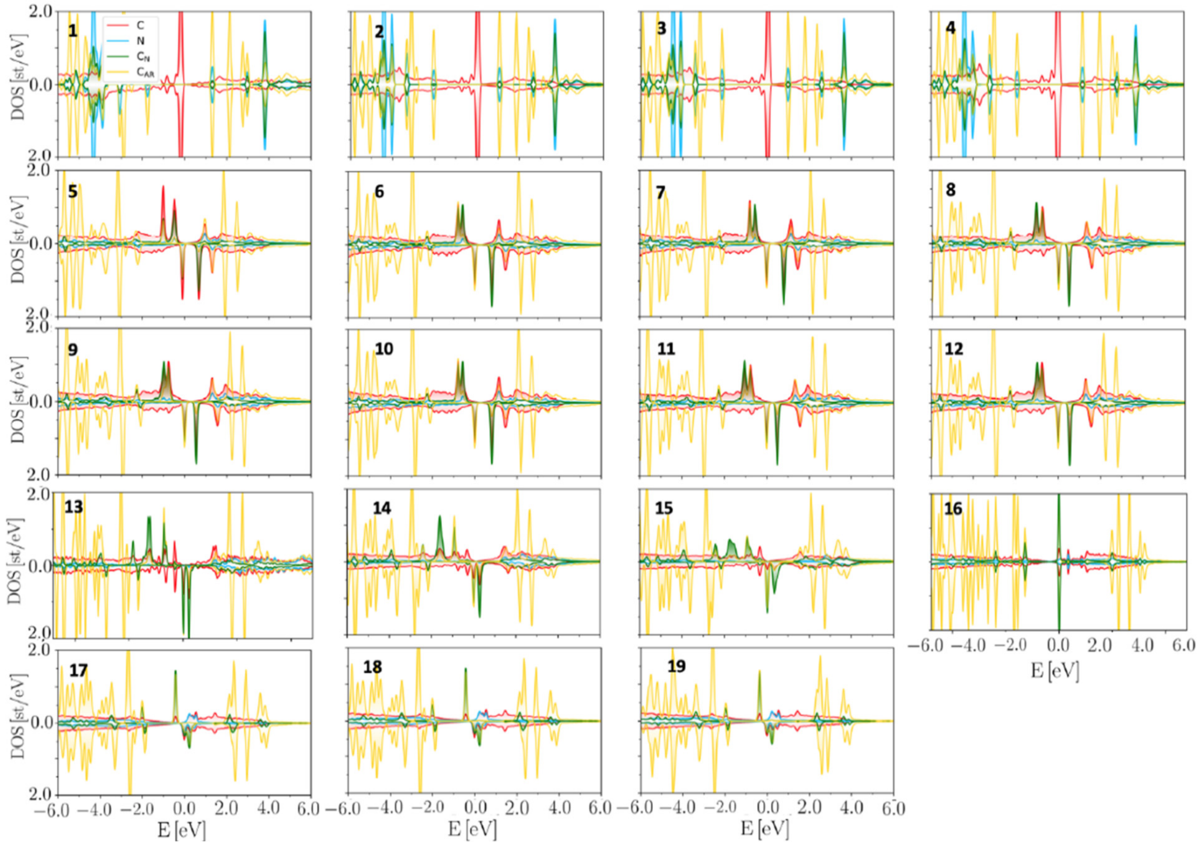

Figure S2. The partial densities of states (PDOS) for the 19 reaction steps generated by the nudge elastic band (NEB) method for the process of 4-cyan-4'-pentylbiphenyl (5CB) into a graphene monovacancy.
